# The anti-fibrotic agent nintedanib protects chondrocytes against tumor necrosis factor-ɑ (TNF-ɑ)-induced extracellular matrix degradation

**DOI:** 10.1080/21655979.2022.2036899

**Published:** 2022-02-14

**Authors:** Chuankun Wang, Lizhe Qu

**Affiliations:** a Department of Orthopedics, Zhoupu Hospital, Pudong New Area, Shanghai, China; b Department of Anesthesiology, Shanghai Traditional Chinese Medicine Hospital Affiliated to Shanghai University of Traditional Chinese Medicine, Shanghai, China

**Keywords:** Osteoarthritis, chondrocytes, nintedanib, oxidative stress, inflammation, extracellular matrix (ECM), PKA/CREB signaling pathway

## Abstract

Osteoarthritis is an inflammatory disease of the musculoskeletal system characterized by damaged articular cartilage. Nintedanib is an oral triple kinase inhibitor with anti-fibrotic and anti-inflammatory properties. Thus, we hypothesized that nintedanib might exert a protective effect in chondrocytes and it could be meaningful to repurpose the drug for osteoarthritis. In this study, we aimed to investigate the potential effects of nintedanib on TNF-α-induced cellular injury in CHON-001 chondrocytes. The results show that nintedanib ameliorated TNF-α-induced reactive oxygen species (ROS) production and reduced glutathione (GSH) decrease. Nintedanib reduced the production of pro-inflammatory cytokines interleukin-6 (IL-6) and interleukin-1β (IL-1β) in TNF-α-induced CHON-001 chondrocytes. Nintedanib restored TNF-α caused decreased expression levels of Col II and sry-type high-mobility-group box-9 (SOX-9) in CHON-001 chondrocytes. Moreover, nintedanib ameliorated the TNF-α-caused impairment of protein kinase A/cAMP-response element-binding protein (PKA/CREB) signaling pathway as revealed by the decreased PKA RI expression and increased p-CREB in CHON-001 cells. Inhibition of PKA by H89 abolished the effects of nintedanib on SOX-9 and Col II expression. Taken together, nintedanib presented protective effects on TNF-α-induced oxidative stress, inflammation, and ECM damage in CHON-001 chondrocytes. Mechanically, the effect of nintedanib is associated with the PKA/CREB pathway. These data imply that the anti-fibrotic agent nintedanib may have a potential therapeutic application for osteoarthritis.

## Graphical Abstract

**Figure:**
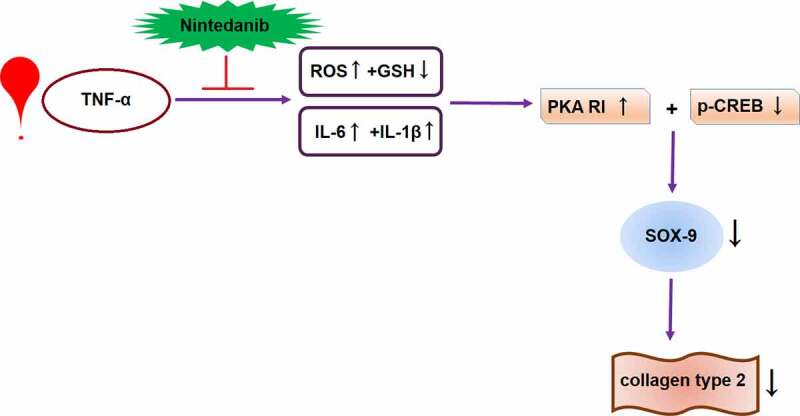

## 1. Introduction

Osteoarthritis is the most common chronic disease of the musculoskeletal system nowadays and its incidence is on the rise [1]. Osteoarthritis is an inflammation-associated condition that leads to progressive functional decline and loss of life quality, resulting in a high burden of health care and society costs [2]. Osteoarthritis is mainly characterized by damage to articular cartilage due to biochemical and biomechanical changes in several joint tissues, which can lead to irreversible degradation of the cartilage [3].

The cartilage matrix comprises two major components: type II collagens and aggrecans. Type II collagens form the structure of cartilage, and aggrecans intersperse in the collagen matrix. Chondrocytes are the predominant cell type within articular cartilage and are responsible for regulating the specialized extracellular matrix (ECM) by synthesizing ECM components and ECM degrading enzymes, thereby maintaining normal articular cartilage structure [4,5]. Under pathological conditions, such as inflammatory conditions and abnormal mechanical loading or injury, chondrocytes undergo oxidative stress, inflammation, and apoptosis [6]. In addition, chondrocytes produce more catabolic factors, which are involved in cartilage degradation. Subsequently, the cartilage integrity is disrupted, eventually leading to the complete loss of articular cartilage [7]. Therefore, changes in chondrocytes’ behaviors are associated with the progression of osteoarthritis.

The current treatment of osteoarthritis is only limited to medication that reduces pain and improves mobility through different approaches. The future treatment strategy of osteoarthritis could face new challenges and new promises. Several new technology and innovative approaches have been reported. For example, nanoparticles formulated with fungal compounds and their analogues have the potential to be developed as immunotherapy agents for immune diseases [8,9]. The recent development of the small extracellular vesicles from human umbilical cord-derived mesenchymal stem cells exhibited the capacity for osteogenic, and chondrogenic differentiation [10]. Meanwhile, the repurposing of existing medication provides innovative approaches to seek anti-osteoarthritic agents.

Nintedanib is an oral triple kinase inhibitor that has been used for the treatment of idiopathic pulmonary fibrosis (IPF) targeting pro-fibrotic pathways [11]. In addition to its antifibrotic activity, it also shows anti-inflammatory activity in a pulmonary fibrosis animal model [12]. Nintedanib attenuates airway inflammation and remodeling in a mouse model of acute asthma through regulating airway eosinophilic inflammation and Th2 cytokines and airway hyper-reactivity [13]. In addition, there is increasing evidence that it also has anti-inflammatory properties outside of the lung tissue. Particularly, in a mouse model of rheumatoid arthritis (RA)-associated interstitial lung disease, nintedanib reduces pulmonary fibrosis and blocks the development of arthritis [14].

The anti-fibrotic and anti-inflammatory properties present ample evidence that this agent could be a potential candidate for drug repurposing. We hypothesized that nintedanib might exert a protective effect on osteoarthritis. In this study, we aimed to investigate the effects of nintedanib on TNF-α-induced inflammatory damage in CHON-001 chondrocytes.

## 2. Materials and methods

### 2.1 Cell culture and treatment

Human chondrocytes cell line CHON-001 (ATCC, Rockville, MD) were maintained in DMEM (Thermo, Waltham, MA) with 10% FBS (Thermo Fisher Scientific, USA) under a 5% CO_2_ atmosphere with 95% humidity [15]. We obtained the cell treatment reagents human TNF-α and Nintedanib from Sigma-Aldrich (St. Louis, USA). CHON-001 cells were stimulated with TNF-α (10 ng/mL) with or without the presence of nintedanib (15 μM) for 24 h. For the PKA inhibitor experiment, the cells were treated with PKA inhibitor H89 (10 μM) for 24 h.

### 2.2 Measurement of lactate dehydrogenase (LDH) release

CHON-001 cells were treated with a series of concentrations of nintedanib (0, 0.75, 1.5, 7.5, 15, 75, and 150 μM) for 24 h. Cytotoxicity of the drug on CHON-001 cells was assessed using the LDH release assay, which was performed using a commercially available kit (Beyotime, Shanghai, China) to detect the LDH release level in the medium according to the manufacturer’s protocol [16,17].

### 2′,7′-Dichlorofluorescin diacetate (DCFH-DA) staining

To measure ROS generation in CHON-001 cells, the probe DCFH-DA (10 μM; Beyotime) was added to the cells for 25 min incubation. Cells were then detected with a fluorescence microscope (Zeiss, Germany) and the fluorescence intensity was analyzed using a fluorescence microplate reader (excitation/emission 488/525 nm) (BioTek Instruments, Inc., USA).

### 2.4 Measurement of reduced GSH

The CHON-001 cells were treated as described above, and the cellular GSH content was measured with a commercial GSH commercial assay kit (Beyotime, China) as reported. In brief, the cells were equilibrated with cold PBS and then lysed with the lysis buffer from the kit. The lysate was centrifuged, and the supernatant samples were collected to measure the protein concentration using a BCA protein assay kit (Thermo Fisher Scientific, USA). The reduced GSH level was measured according to the manufacturer’s instructions.

### 2.5 RT-PCR

Total RNA of CHON-001 cells extracted by TRIzol reagent (Thermo) was used to analyze the mRNA levels of *IL-6, IL-1β*, collagen type II (*Col II*), and *SOX-9*. PrimeScript RT reagent kit and SYBR Premix Ex Taq II kit (Takara, Dalian, China) were respectively used for the reverse transcription and PCR reactions. Data were analyzed using the 2^−ΔΔCT^ method.

### 2.6 ELISA

After indicated treatments, samples of the supernatant from CHON-001 cells were collected. The collected samples were quantified using a BCA protein assay kit (Thermo Fisher Scientific, USA). The amount of secreted IL-6 and IL-1β was measured using corresponding ELISA kits (Jiancheng BioTech., Nanjing, China). The assay procedure was performed according to the manufacturer’s instructions.

### 2.7 Western blot

Total proteins of CHON-001 cells extracted with RIPA lysate buffer (Solarbio, Beijing, China) were used for the determination of protein levels of Col II, SOX-9, PKARI, and p-CREB using Western blot analysis [16,17]. The primary antibodies against indicated target proteins as well as HRP-linked IgG secondary antibodies were obtained from Santa Cruz Biotechnology (Santa Cruz, CA). The intensity of protein bands was analyzed using Image Lab Software (Bio-Rad, Hercules, CA).

### 2.8 Statistical analysis

All results were repeated three times and the data were analyzed with the usage of GraphPad Prism 8 statistical software. All values were presented as mean ±  standard error of mean (S.E.M). Comparisons of data among multiple groups were performed using analysis of variance (ANOVA), followed by Tukey’s post-hoc test. *P* < 0.05 was considered statistically significant.

## 3. Results

In the current study, our data show that nintedanib ameliorated the TNF-α-induced oxidative stress and pro-inflammatory cytokine production in CHON-001 chondrocytes. Nintedanib ameliorated TNF-α-caused decreased synthesis of extracellular matrix Col II and chondrogenesis regulator SOX-9. Mechanistically, nintedanib ameliorated the TNF-α-caused impairment of the PKA/CREB signaling pathway.

### 3.1 Cytotoxicity of nintedanib in CHON-001 chondrocytes

**Figure 1.**
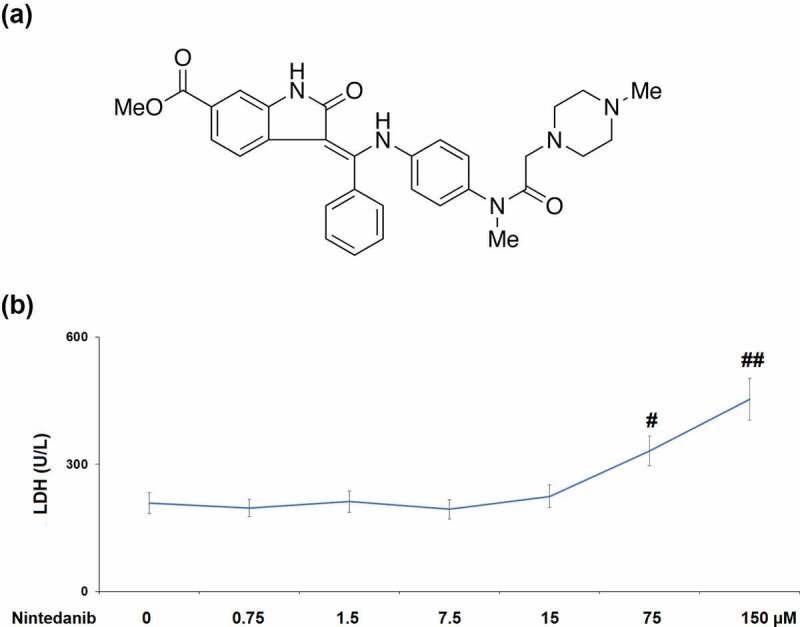
Cytotoxicity of Nintedanib in CHON-001 chondrocytes. Cells were treated with 0.75, 1.5, 7.5, 15, 75, and 150 μM for 24 hours. (a). Molecular structure of Nintedanib; (b). Cytotoxicity was assayed by measuring LDH release (#, ##, P < 0.05, 0.01 vs. Vehicle group).

To validate whether nintedanib (Structure in Figure 1(a)) had cytotoxicity in CHON-001 cells, LDH release was assessed after incubating with 0.75, 1.5, 7.5, 15, 75, and 150 μM nintedanib for 24 h. As shown in Figure 1(b), CHON-001 cells treated with 75 or 150 μM nintedanib exhibited a significant increase in LDH release with a 1.6- or 2.2-fold change. Therefore, 7.5 and 15 μM nintedanib were used for the subsequent experiments.

### 3.2 Nintedanib ameliorated TNF-α-induced oxidative stress in CHON-001 chondrocytes

**Figure 2.**
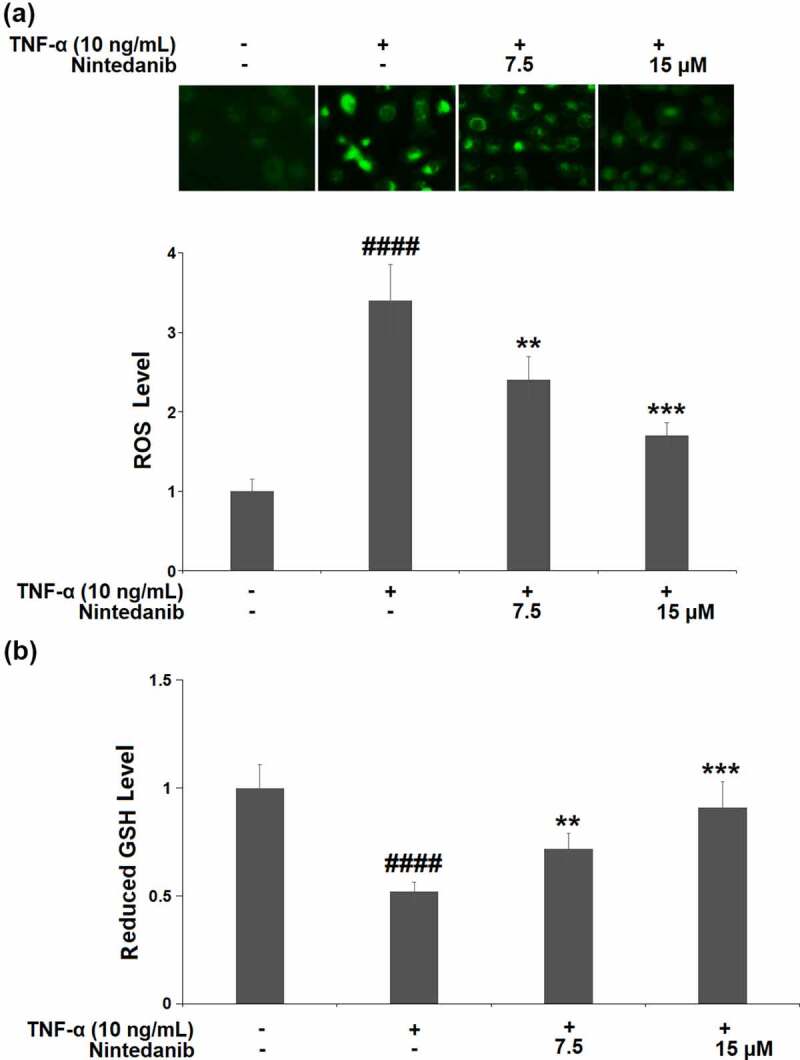
Nintedanib ameliorated TNF-α-induced oxidative stress in CHON-001 chondrocytes. Cells were stimulated with TNF-α (10 ng/mL) with or without Nintedanib (7.5, 15 μM) for 24 hours. (a). Intracellular levels of ROS; (b). The levels of reduced GSH (####, P < 0.0001 vs. Vehicle group; **, ***, P < 0.01, 0.001 vs. TNF-α group).

TNF-α-induced CHON-001 cells yielded a 3.4-fold increase in intracellular levels of ROS. The increased ROS level was markedly reduced by nintedanib (7.5 and 15 μM) treatment with 29.4% and 50.0% reductions, respectively (Figure 2(a)). GSH (reduced form) is the most important hydrophilic antioxidant that protects chondrocytes from ROS-induced injury. We also measured the cellular GSH levels. CHON-001 cells under TNF-α induction exhibited a significant reduction (0.52-fold change) in the level of reduced GSH, which was mitigated by nintedanib (7.5 and 15 μM) (Figure 2(b)).

### 3.3 Nintedanib inhibited TNF-α- induced expressions of IL-6 and IL-1β in CHON-001 chondrocytes

**Figure 3.**
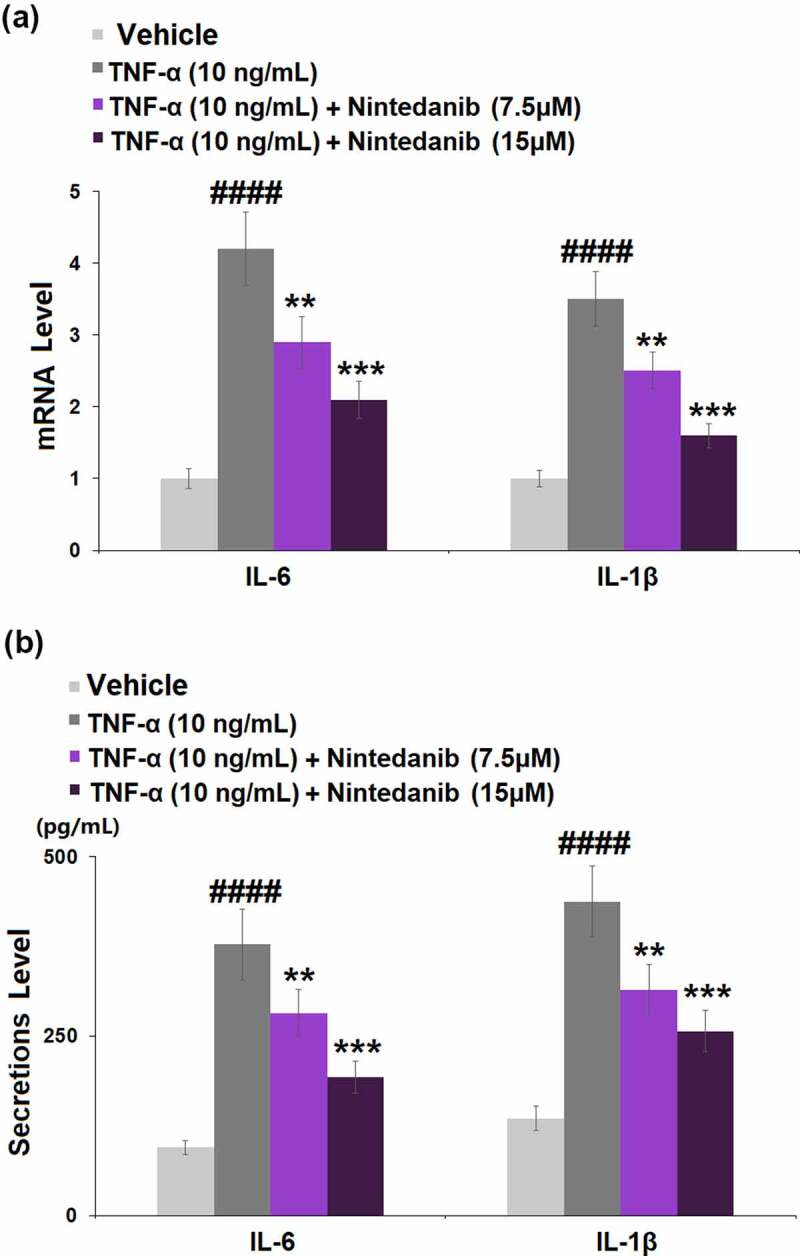
Nintedanib inhibited the TNF-α-induced expression of pro-inflammatory cytokines such as IL-6 and IL-1β. Cells were stimulated with TNF-α (10 ng/mL) with or without Nintedanib (7.5, 15 μM) for 24 hours. (a). mRNA of IL-6 and IL-1β; (b). Secretions of IL-6 and IL-1β (####, P < 0.0001 vs. Vehicle group; **, ***, P < 0.01, 0.001 vs. TNF-α group).

The results in Figure 3(a) show that exposure to TNF-α (10 ng/mL) caused a significant increase in the mRNA levels of *IL-6* (4.2-fold) and *IL-1β* (3.5-fold). Pretreatment with nintedanib (7.5 or 15 μM) led to obvious decreases in both mRNA levels of *IL-6* (0.7- and 0.5-fold) and *IL-1β* (0.7- and 0.4-fold) when compared with cells stimulated with TNF-α only. Consistently, stimulation with TNF-α effectively induced the secretion of IL-6 (4.0-fold) and IL-1β (3.2-fold), whereas nintedanib (7.5 or 15 μM) intervention reversed the TNF-α-caused induction of IL-6 and IL-1β secretion (Figure 3(b)).

### 3.4 Nintedanib restored the decrease in the expression of Col II

**Figure 4.**
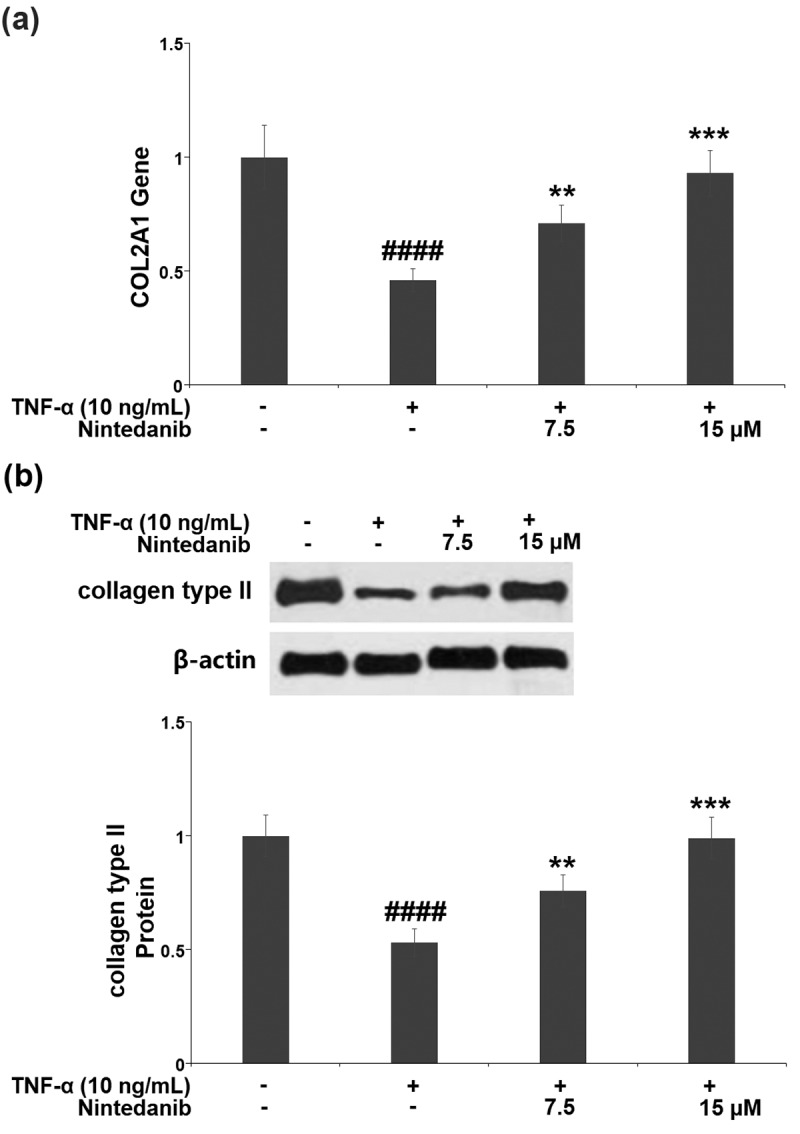
Nintedanib restored the decrease in the expression of collagen type II. (a). Gene of COL2A1; (b). Protein of collagen type II (####, P < 0.0001 vs. Vehicle group; **, ***, P < 0.01, 0.001 vs. TNF-α group).

Results in Figure 4(a) indicate that TNF-α induced a significant decrease (0.46-fold) in the mRNA level of Col II in CHON-001 chondrocytes, while the decreased *Col II* mRNA was elevated 1.5- or 2.0-fold after treatment with nintedanib (7.5 or 15 μM). Consistent with the changes in its mRNA level, the protein level of Col II was reduced by 47.0% in TNF-α-stimulated cells, which was attenuated by 7.5 or 15 μM nintedanib (Figure 4(b)).

### 3.5 Nintedanib prevented TNF-α- induced reduction of SOX-9

**Figure 5.**
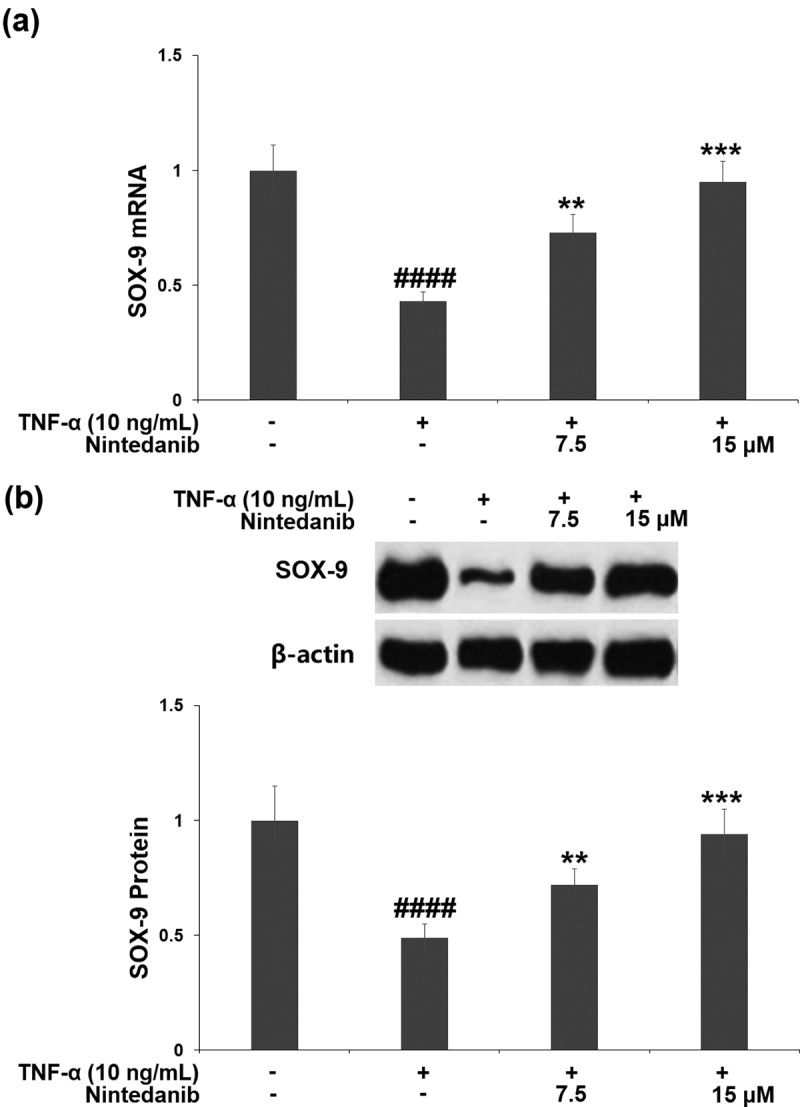
Nintedanib prevented the TNF-α-induced reduction of SOX-9. (a). mRNA levels of SOX-9; (b). Protein levels of SOX-9 (####, P < 0.0001 vs. Vehicle group; **, ***, P < 0.01, 0.001 vs. TNF-α group).

After TNF-α induction, the mRNA level of *SOX-9* in CHON-001 chondrocytes was significantly decreased by 57.0%. The *SOX-9* mRNA level was increased by 1.7- and 2.2-fold after treatment with 7.5 or 15 μM nintedanib (Figure 5(a)). A similar response was found in its protein level, the TNF-α-caused reduction (51.5%) in the SOX-9 protein level was elevated by 7.5 or 15 μM nintedanib (1.5- and 1.9-fold) (Figure 5(b)).

### 3.6 The protective effects of nintedanib are associated with the PKA/CREB/SOX-9 signaling pathway

**Figure 6.**
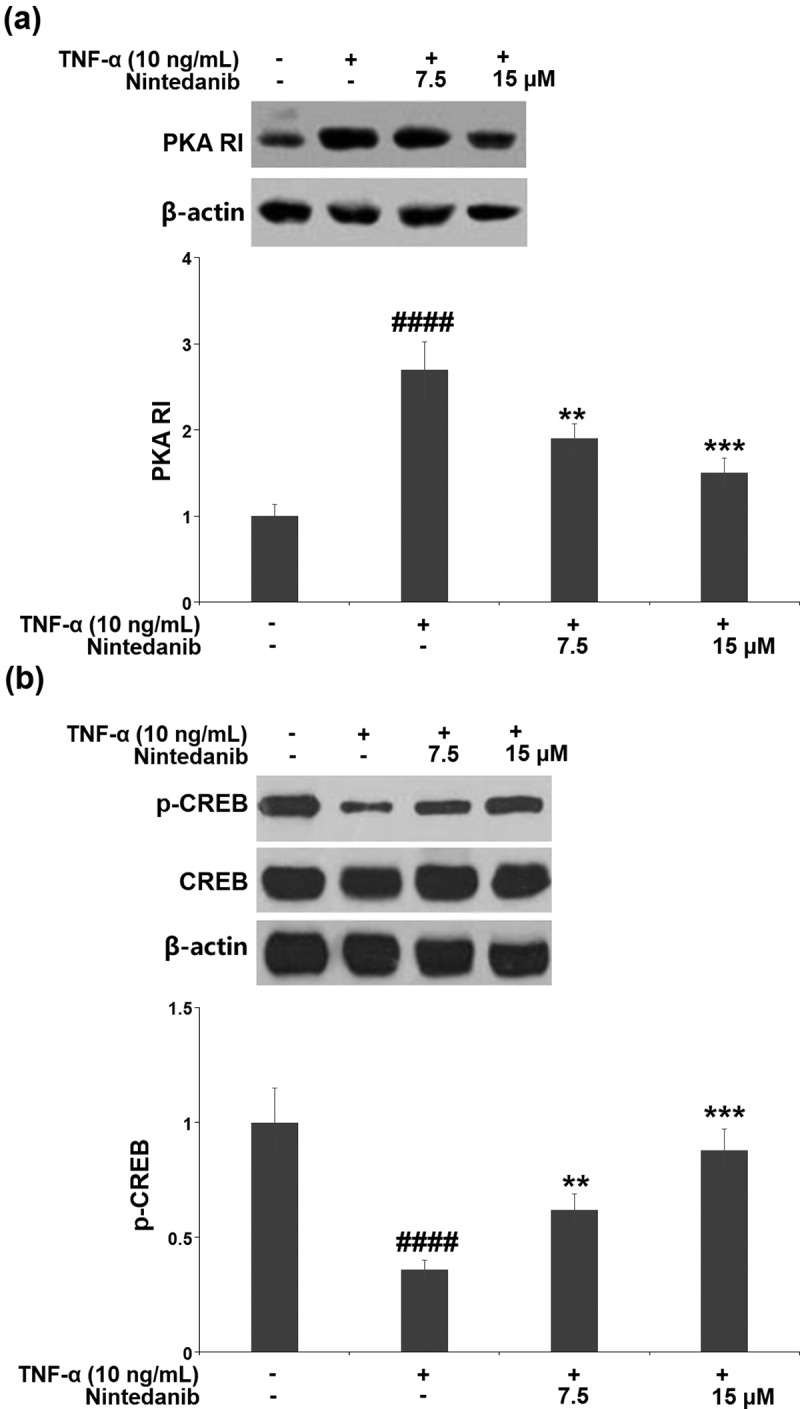
Nintedanib restored the levels of PKA RI and p-CREB against TNF-α. Cells were stimulated with TNF-α (10 ng/mL) with or without Nintedanib (7.5, 15 μM) for 6 hours. (a). The expression of PKA RI; (b). The levels of p-CREB (####, P < 0.0001 vs. Vehicle group; **, ***, P < 0.01, 0.001 vs. TNF-α group).

Exposure of CHON-001 chondrocytes to TNF-α resulted in a significant increase in PKA RI expression (2.7-fold) and a decrease in p-CREB (0.36-fold). Pretreatment with 7.5 or 15 μM nintedanib reversed these changes in PKA RI expression and p-CREB (Figure 6(a,b)).

**Figure 7.**
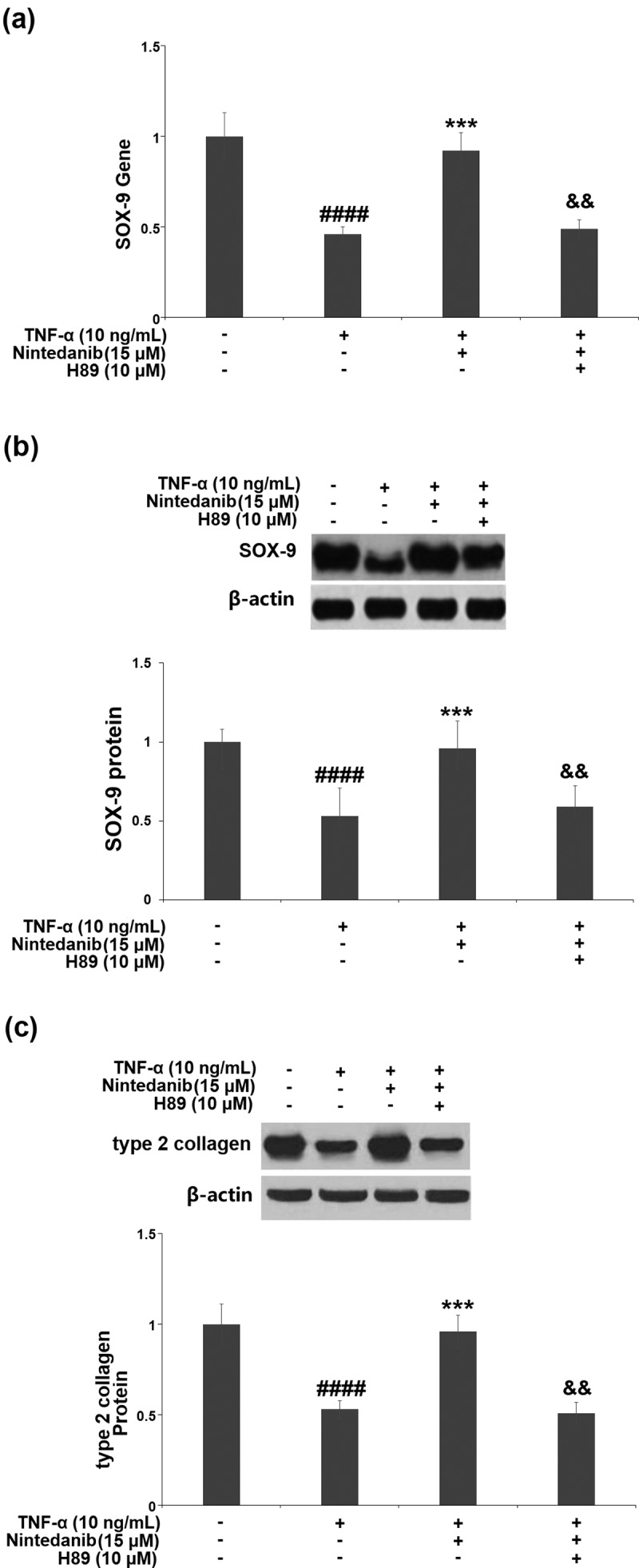
Blockage of the PKA/CREB signaling with H89 abolished the protective effects of Nintedanib against TNF-α- induced reduction of SOX-9 and collagen type II. Cells were stimulated with TNF-α (10 ng/mL) with or without Nintedanib (15 μM) and H89 (10 μM) for 24 hours. (a). Gene levels of SOX-9; (b). Protein levels of SOX-9; (c). Protein levels of type 2 collagen (####, P < 0.0001 vs. Vehicle group; **, ***, P < 0.01, 0.001 vs. TNF-α group).

RT-PCR results show that the nintedanib (15 μM)-induced increase in *SOX-9* mRNA was reversed by H89 (10 μM) (Figure 7(a)). Meanwhile, the result of Western blot shows that nintedanib also induced SOX-9 protein but was attenuated when the PKA inhibitor H89(10 μM) was present (Figure 7(b)). Further, the Western blot analysis indicated that the inductive effect of nintedanib (15 μM) on Col II expression was restrained by H89 (10 μM) (Figure 7(c)).

## 4. Discussion

Along with the anti-fibrotic and anti-inflammatory capacity in the lung, nintedanib also exerts anti-inflammatory and anti-oxidative effects in other tissues. Notably, Nintedanib is beneficial in attenuating hepatic necrosis, inflammation, and fibrosis in the CCl_4_-induced hepatic injury model in mice [18]. Nintedanib attenuates the expression of cytokines/chemokines, including TNF-α, IL-1β, and IL-6, monocyte chemoattractant protein-1 (MCP-1) and prevents the macrophages infiltration to the injured peritoneum in chlorhexidine gluconate (CG)-induced peritoneal fibrosis [19]. Nintedanib ameliorates imiquimod-induced psoriasis in mice through improving keratinocyte homeostasis via inhibiting hyperproliferation, regulating the expression of proinflammatory factors, and promoting apoptosis [20]. Nintedanib attenuates unilateral ureteral obstruction-elicited expression of proinflammatory cytokines and macrophage infiltration in the kidney [21]. Moreover, nintedanib presents a potential anti-oxidative effect in chronic lung allograft dysfunction through downregulation of ROS and transglutaminase-2 (TGM-2) [22]. Nintedanib exerts a beneficial effect on specific markers of systemic oxidative stress and inflammation in idiopathic pulmonary fibrosis (IPF) patients [23]. These data support that Nintedanib possesses a broad spectrum of anti-inflammatory and anti-oxidative benefits in various cells.

Osteoarthritis is a degenerative joint disease with irreversible structural and functional changes in cartilage. During the development of osteoarthritis, oxidative stress and inflammation are interdependent, leading to ECM degradation and joint dysfunction [24]. Oxidative stress and inflammatory response are critical events that contribute to the progression of osteoarthritis [25,26]. As the sole cell type in cartilage tissue, chondrocytes are exposed to high inflammation and oxidative stress-induced micro-environment, the quiescent state of metabolic balance is impaired, and the balanced function to synthesis and degradation of extracellular matrix is disturbed. To remedy the elevated inflammatory status, we sought the drug repurposing study to validate the effect of nintedanib in cytokine elicited chondrocytes. Our results indicate that nintedanib ameliorated TNF-α-induced production of key mediators of oxidative stress and inflammation in CHON-001 chondrocytes. Our tests prove that nintedanib restored the decrease in the expression of Col II, which is a major component of ECM. SOX-9, a member of the Sry-related high mobility group box (SOX) family, is an anabolic transcription factor that plays an important role in the development of cartilage [27]. SOX-9 is expressed in chondrocytes and contributes to chondrogenesis by activating cartilage-specific genes [28,29]. Since SOX-9 plays an essential role in regulating chondrocytes, its dysregulation is likely to contribute to the pathology of OA. A previous study has shown that SOX-9 has been found to be downregulated in osteoarthritic cartilage [30]. Here, we demonstrate TNF-α-induced reduction of SOX-9 expression in CHON-001 chondrocytes. Furthermore, its expression was mitigated by nintedanib treatment. These data suggest the nintedanib treatment modulates the inflammation associated with SOX-9 reduction, and that SOX-9 could be a mediator of nintedanib in chondrocytes. Our data confirm that nintedanib restrained TNF-α-induced oxidative stress, inflammation, and ECM damage in CHON-001 chondrocytes, implying its beneficial effect in chondrocytes.

The PKA/CREB signaling pathway has been shown to be involved in chondrogenesis regulation. PKA, a key cellular target for cAMP, and PKA contains two catalytic and two regulatory subunits [31]. After binding to cAMP through its regulatory subunits, the catalytic subunits become active and thereby phosphorylate the specific residues (serine or threonine) on the substrate proteins [31]. CREB is a well-known major nuclear target of PKA and is critical for cellular differentiation, proliferation, and adaptive responses [32,33]. Studies on the PKA/CREB signaling pathway have shown that it is involved in the pathogenesis of various inflammatory diseases including osteoarthritis [34–36]. Another study showed that Liraglutide ameliorated inflammation in a rat osteoarthritis model through the activation of the PKA/CREB signaling pathway [37]. A most recent study reveals that Follicle-stimulating hormone (FSH) modulates cartilage ECM metabolism in chondrocyte-like ATDC5 cells by targeting the PKA/CREB/SOX9 pathway [38]. It has been known that CREB directly regulates SOX-9 expression by binding to its promoter, but PKA phosphorylates the SOX-9 protein by epigenetic regulation [39]. Based on these evidences, we next assessed whether the PKA/CREB pathway participates in the protective effects of nintedanib on TNF-α-induced CHON-001 chondrocytes. We found that nintedanib restored the levels of PKA RI and p-CREB in CHON-001 cells after TNF-α stimulation. Additionally, inhibition of PKA by H89 abolished the effects of nintedanib on SOX-9 and Col II expression, implying that the protective effects of nintedanib on ECM damage were mediated by the PKA/CREB signaling pathway.

In light of the above discussion, the limitations of the study have to be addressed. First, the current studies were all performed in immortalized chondrocyte CHON-001 cells. Although often used as one of the well-established osteoarthritis models *in vitro*, this cell line is not equivalent to primary chondrocytes in humans [17]. To validate the current findings, future tests on primary chondrocytes from human cartilage or animal models are necessary. Secondly, the pharmacological mechanism of nintedanib in chondrocytes and cartilage tissue remains to be fully elucidated. So far, it is unclear how nintedanib exerts its effect on the inflammatory mediators and ROS generation. It may involve the regulation of the PKA/CREB/SOX-9 pathway, but could also have other cellular pathways. Furthermore, the optimal dosage of nintedanib in chondrocytes remains to be tested for its best effect.

## Conclusion

In conclusion, we provided evidence that the anti-fibrosis drug nintedanib possesses a protective effect against TNF-α-induced oxidative stress, inflammation, and ECM damage in CHON-001 chondrocytes. Mechanistically, the biological function of nintedanib is partially attributed to the regulation of the PKA/CREB/SOX-9 signaling. These findings indicate that the repurposing of nintedanib might be meaningful for developing a potential therapeutic strategy for osteoarthritis.

## Data Availability

The data that support the findings of this study are available from the corresponding author upon reasonable request.

## Highlights

1. miR-3646 was upregulated in LUAD tissues and cells, whereas SORBS1 was downregulated.

2. miR-3646 overexpression stimulated the proliferation, adhesion, and tumor growth of LUAD cells and inhibited apoptosis.

3. SORBS1 was identified as a target gene downstream of miR-3646.

4. SORBS1 overexpression suppressed the malignant phenotypes of LUAD cells, which was restored by upregulation of miR-3646.

5. miR-3646 overexpression activated the JNK signaling pathway in LUAD cells, whereas SORBS1 overexpression inhibited it.
